# Classification in Workhouses.—How to Make It Effective

**Published:** 1896-08-29

**Authors:** 


### Modern Sociology.

### CLASSIFICATION IN WORKHOUSES.—HOW TO MAKE IT EFFECTIVE.

A few months ago we had cause to study closely the precise wording of Guy's will and the Act of Parliament which was based upon it. This close examination revealed the fact that the late Thomas Guy's views of hospital administration embraced all, or nearly all, which the most modern reformers and experts could suggest for the promotion of efficient administration. It follows that the administrative and other troubles of Guy's Hospital, of which the public has heard so much in recent days, would have been avoided, and the abuses connected with them largely, if not entirely, prevented, had Thomas Guy's views prevailed in the administration of the affairs of this hospital from the date of the opening ceremony to the present day. Similarly it may be urged with some force that the action of the Local Government Board, in directing the attention of guardians of the poor all over the country to one of the General Consolidated Orders made fifty years ago, as the authority upon which they should base their arrangements for the classification of aged and infirm inmates of workhouses, points to the conclusion that guardians have always had the power, but few of them have had the will, to provide adequately for the poor of all classes. Indeed, we would go further, and say that these two instances of the wisdom and foresight of our forbears prove to demonstration the need which exists in the present day for a codification of all the powers entrusted to the Local Government Board and to guardians of the poor on the one hand, and to the managers of great and ancient endowments, and of many public institutions on the other. It appears from No. 48 of the General Consolidated Orders of the 24th July, 1847, that guardians have for nearly fifty years been empowered to classify certain classes of workhouse residents with reference to their moral character, behaviour, and previous habits, and on such other grounds as may seem to them expedient. It follows that many if not all the objections which the respectable poor rightly have to enter the workhouse would have been non-existent had the guardians everywhere throughout the country discharged their duties with conscientious thoroughness and a due regard to the best interests of the poor. With certain exceptions classification has not been attempted at all, and the exceptions emphasise the callous disregard of duty which permeates the kingdom of Bumble, for we have been unable to find a single instance where the classification has been carried out either with thoroughness or with any adequate regard for the interests of these poor people, which such a system should protect and encourage. It is therefore useless to expect that the recent circular of the Local Government Board will effect the desired end, or that any real protection will be afforded to the respectable poor in workhouses until the present system of administration by boards of guardians has been altered.

What we need at the present time is an Act of Parliament abolishing the local boards of guardians, or limiting their authority to that of sub-committees of a county Poor Law Board, which should exercise authority over each county. At the present time the country contains an adequate number of workhouses, and abundance of accommodation for the aged and infirm poor of every parish throughout England. The whole of the poor law buildings within each county area should be handed over to the control of the new county poor law boards. These county poor law boards could then classify the various buildings according to the needs and requirements of all classes of the poor in the area under their control. The accommodation is ample, as we have said, many of the existing workhouses providing accommodation far in excess of the demands made upon the guardians who control them. Hence the new county poor law boards could readily set aside the most suitable buildings for the respectable poor and could make such arrangements for their comfort and accommodation, without additional cost, that the prejudice now justly attaching to workhouses would largely disappear, and justice would at last be done to the really deserving who have been left stranded in the battle of life despite their respectability and good moral character and through no fault of their own. Other buildings within the area could be assigned to the immoral, the thriftless, confirmed ne'er-do-weels, and the wastrels and abandoned poor who at present find their way to the workhouse as their natural home.

Such a modification of the existing poor law system as is here proposed would have far-reaching consequences. It would enable poor law relief to be so administered as to make its restraining influence of the utmost possible value in dealing with the least worthy classes of the poorer population. The conditions of workhouse residents, so far as these classes are concerned, could be made to possess an educational value, discipline could be adequately enforced, and restraints and punishments where needed could be made effective from the circumstance that the offenders would gradually come to realise that their influence for harm was destroyed, and that it was to their interest to devote all their energies thenceforward to the improvement of their own character and condition. Further and better still, the worthy and deserving poor who entered the workhouse would be freed from evil surroundings and companions; they could then be made to feel that they possessed the sympathy of their more fortunate countrymen, who wished them torealize that they recognised that circumstances had been against them, and that there was no stigma attaching to this class of the poor in workhouses; their friends would be encouraged to visit them, they would be at liberty to visit their friends, their health would be promoted by healthy outdoor occupation were they able to pursue it by the cultivation of the grounds attached to the workhouse, and the last few years of their life would thus prove, it may be hoped, happy and beneficial in every respect.

